# Impact of American Thyroid Association’s Revised Cancer Management Guidelines on Thyroid Cancer Incidence Trends: A Retrospective Cohort Study, 2000–2020

**DOI:** 10.3390/jcm14010028

**Published:** 2024-12-25

**Authors:** Pierre Fwelo, Natalia I. Heredia, Ruosha Li, Ayrton Bangolo, Vignesh K. Nagesh, Simcha Weissman, Xianglin L. Du

**Affiliations:** 1 Department of Epidemiology, School of Public Health, University of Texas Health Science Center at Houston, 1200 Pressler St., Houston, TX 77030, USA; xianglin.l.du@uth.tmc.edu; 2 Department of Health Promotion and Behavioral Sciences, School of Public Health, University of Texas Health Science Center at Houston, 1200 Pressler St., Houston, TX 77030, USA; natalia.i.heredia@uth.tmc.edu; 3 Department of Biostatistics & Data Science, School of Public Health, University of Texas Health Science Center at Houston, 1200 Pressler St., Houston, TX 77030, USA; ruosha.li@uth.tmc.edu; 4 Department of Hematology and Oncology, John Theurer Cancer Center at Hackensack University Medical Center, Hackensack, NJ 07601, USA; ayrton.bangolo@hmhn.org; 5 Department of Internal Medicine, Palisades Medical Center, North Bergen, NJ 07047, USA; vigneshkrishnan.nagesh@hmhn.org (V.K.N.); simcha.weissman@hmhn.org (S.W.)

**Keywords:** thyroid cancer, incidence, trends, racial disparities, sociodemographic

## Abstract

**Background:** The past four decades have seen a steady increase in thyroid cancer in the United States (US). This study investigated the impact of the American Thyroid Association (ATA)’s revised cancer management guidelines on thyroid cancer incidence trends and how the trends varied by socioeconomic, histologic, geographic, and racial and ethnic characteristics from 2000 to 2020. **Methods:** We used data from the Surveillance, Epidemiology, and End Results (SEER) database to identify thyroid cancer cases diagnosed among US patients between 2000 and 2020. We employed joinpoint regression software to fit, assess, and compare thyroid cancer incidence trends over time stratified by socioeconomic status (SES), histologic type, geographic location, and race/ethnicity. **Results:** Between 2000 and 2009, there was an average annual increase of 5.8% in thyroid cancer incidence (average annual percent change (AAPC): 5.8, *p* < 0.05). Subsequently, there was a modest rise (AAPC: 1.1, *p* < 0.05) from 2010 to 2015, followed by a significant annual decrease of 4.8% from 2016 to 2020 (AAPC: −4.8, *p* < 0.05). The joinpoint regression models identified prominent inflection points around 2009 and 2015, aligning with the years of the ATA’s cancer management revisions. These intricate dynamics in thyroid cancer incidence trends from 2000 to 2020 were shaped by SES and histologic, geographic, and racial/ethnic factors. **Conclusions:** Thyroid cancer incidence trends over the past two decades can be partially explained by the changes in thyroid cancer screening and management recommendations. These findings underscore the importance of cancer management strategies and highlight the need for targeted interventions to address disparities in thyroid cancer incidence across minority demographic groups.

## 1. Background

Thyroid cancer is the most common endocrine malignancy in the United States (US), with approximately 979,295 people living with it in 2021 and 44,020 new cases expected to be diagnosed in 2024 [1,2]. Moreover, it is one of the most rapidly increasing cancers in the US, with an incidence rate of 13.7 new cases per 100,000 persons in 2020, compared with only 5.9 cases per 100,000 in 1992 [1,2]. Understanding the temporal trend of thyroid cancer and the factors associated with this trend is crucial for better cancer detection, management, and control of this disease in the US population.

Race, socioeconomic status (SES), sex, histologic type, geographic location, and stage at diagnosis have been suggested as potential determinants of the observed thyroid cancer incidence increase [3,4,5,6]. Scholars have suggested that the increase in thyroid cancer incidence could be explained by the substantial increase in small thyroid tumors (<4 cm) in White compared with Black patients [7]. In contrast, others have found no difference in the increase in thyroid cancer incidence between White and Black patients [8]. Patients with high SES have also been identified as potential drivers of the incidence trend [5]. These patients tend to have greater awareness of their health status, insurance coverage, and access to advanced diagnostic tools than people with low SES; as a result, they are more likely to be diagnosed with thyroid cancer than those with low SES [5,9,10,11]. Before 1990, physical examination, which has a low sensitivity and specificity, was the primary modality recommended to detect thyroid cancer. In the early 1990s, ultrasound and fine-needle aspiration (FNA) were introduced and adopted as the gold standard modalities to screen thyroid nodules felt during a physical examination. Since then, we have observed a rapid increase in the number of thyroid cancers diagnosed and reported [5]. Researchers have argued that the improvement of diagnostic tools has mainly benefited patients with high SES and those residing in metropolitan or high-SES areas. One study estimated that the annual percent change (APC) of thyroid cancer incidence in high-SES counties increased exponentially between 1980 and 2008. In contrast, the APC in low-SES counties remained unchanged in the same period [5]. Lastly, the increase in thyroid cancer incidence has been suggested to result from the significant increases in papillary thyroid cancer incidence (APC = 4.4%, 95% CI 4.0–4.7%) between 1974 and 2013 compared with the other histologic types that had only marginal changes in their trends [12].

In 2006, the ATA published guidelines to enhance the quality of care for patients with thyroid nodules by providing evidence-based screening, evaluation, and management recommendations [13]. The guidelines emphasized the importance of thyroid sonography in all patients with suspected nodules and the role of FNA biopsy as the procedure of choice for evaluating nodules [13]. However, these guidelines lacked extensive coverage of molecular markers for improved diagnostic accuracy and management considerations for special populations like children and pregnant women. Therefore, they were revised in 2009. The 2009 revisions introduced key updates, including a shift toward biopsy primarily based on nodule size and sonographic features to reduce unnecessary procedures [14]. In addition, the revision introduced a four-tiered cytology classification system to facilitate and improve risk stratification and diagnosis accuracy, particularly in cases of inconclusive initial cytology results. In 2015, they were further revised to incorporate a more refined approach to biopsy based on sonographic patterns with surveys of the cervical lymph followed by size, a six-tiered cytology classification, and specific recommendations for molecular marker usage [15,16]. The 2009 and 2015 revisions in the thyroid cancer management guidelines have significant potential clinical implications in shaping the incidence trends [15,16]. For instance, refining biopsy criteria based on sonographic patterns and individual features can potentially enable more precise identification. Conversely, the revisions can reduce the number of thyroid nodules requiring biopsies.

Numerous articles have investigated the factors associated with the increase in thyroid cancer in the US. However, most of this literature has focused on trends of cases diagnosed until 2008, before the ATA’s thyroid cancer management guideline revisions. Studies on cases diagnosed since 2009 show that thyroid cancer incidence increased steadily from 1992 to 2009, then slowed between 2009 and 2014 [17,18,19]. From 2015 onward, research indicates a stable or declining APC in incidence [17,18,19]. The identified inflection years (2009 and 2015) align with ATA revision years, suggesting that the revision of these guidelines has begun to reduce thyroid cancer incidence trends. There is a scarcity of studies on the impact of the ATA’s thyroid cancer management guidelines implementation or the subsequent revisions on thyroid cancer trends in the US, with only one published paper known to us. Shi et al. (2017) examined the impact of the ATA’s 2009 thyroid cancer management guidelines revision on thyroid cancer trends and found that the APC in thyroid cancer incidence slowed from about 8% between 2000 and 2009 to 3% between 2009 and 2012 [19]. However, their study assessed only the first revision and could not evaluate the effects of the 2015 revisions, as they included data only up to 2012. Additionally, they did not evaluate the direct impact of the 2006 ATA implementation on thyroid cancer trends. Furthermore, it remains unclear whether the influence of race, socioeconomic status (SES), histologic type, and geographic area on thyroid cancer incidence has changed since the guidelines were implemented and revised in 2009 and 2015. Therefore, this study aims to examine the impact of the ATA’s original cancer management guidelines and its revisions on thyroid cancer incidence trends and to determine the temporal, socioeconomic, histologic, geographic, and racial variations in thyroid cancer incidence from 2000 to 2020.

## 2. Methods

### 2.1. Study Design, Data Source, and Patient Selection

We conducted this retrospective cohort study using secondary data of patients with a primary diagnosis of thyroid cancer (International Classification of Disease for Oncology, Third Edition code: C73.9) from 2000 to 2020 through the Surveillance, Epidemiology, and End Results (SEER) database. SEER is a cancer surveillance program supported by the National Cancer Institute, the primary source of reliable incidence and survival data in the US. We used SEER research plus data for 17 registries in the November 2022 submission. SEER 17 registries cover Alaska Natives, Connecticut, Atlanta, greater Georgia, rural Georgia, greater California, San Francisco–Oakland, San Jose–Monterey, Hawaii, Iowa, Los Angeles, New Mexico, Kentucky, Louisiana, Seattle–Puget Sound, New Jersey, and Utah. SEER collects patient and tumor characteristics using multiple channels and data collection techniques, including self-reports, medical records, death certificates linked with the National Death Index data, and quality control measures [20]. A detailed database and data collection description can be found elsewhere [20]. We used SEER*stat software 8.4.0 to identify patients with a lab-confirmed diagnosis of thyroid cancer as the first primary cancer from 2000 to 2020. The study subjects were de-identified, and there was no patient contact; thus, the study was exempted from an Institutional Review Board (IRB)’s approval.

### 2.2. Study Variables

Dependent variables: Age-adjusted incidence rates (number of thyroid cancer cases per 100,000 persons) for thyroid cancer between 2000 and 2020 were the primary outcome of interest. They were calculated by dividing the number of new thyroid cases diagnosed between January 1 and December 31 in the areas covered by SEER 17 registries by the population estimates for the counties in those areas each year in the US. Detailed descriptions of how these rates were calculated can be found elsewhere [21,22,23,24]. These rates were adjusted to the 2000 US standard population by age.

Independent variables: In this study, we had five independent variables: Time period, SES, histologic type, geographic location, and race/ethnicity. Time period was classified based on the year at diagnosis into 2000–2009, 2010–2015, and 2016–2020 using ATA revision years as cut points. SES was categorized into three groups: low (0–10,972), middle (10,973–11,513), and high (11,514–11,870) SES tertile, based on the Yost index that has been created by other investigators [25,26]. The Yost index is a composite metric of SES based on principal component of census block groups within county-level variables such as median household income adjusted for inflation, median house value, median rent, percent below 150% of the poverty line, education index, percent working class, and percent unemployed [25,26]. Histologic type was classified into papillary thyroid cancer (PTC), follicular (FTC), medullary (MTC), anaplastic (ATC), and other. Geographic location was categorized into metropolitan and non-metropolitan. Lastly, race/ethnicity was classified into non-Hispanic (NH) White, NH Black, Hispanic, and NH other patients.

Covariates: Cancer characteristics and sociodemographic characteristics selected for this study were based on previous studies [5,27,28]. The sociodemographic characteristics included age (< 40, 40–49, 50–59, 60–69, 70–79, and 80+), sex (male/female), and marital status (married/domestic partner, divorced/separated, widowed, and never married). Cancer characteristics included tumor size (0–1, 1.1–2.0, 2.1–4.0, and more than 4 cm) and tumor stage (early (i.e., localized)/advanced (i.e., regional and distant)). All variables were assigned an unknown category where applicable.

### 2.3. Statistical Analysis

We performed descriptive analyses to examine the characteristics of the cohorts stratified by race/ethnicity. We performed Pearson’s chi-square test to determine statistically significant differences between groups. We used SEER*Stat version 8.40 to compute annual age-adjusted incidence rates stratified by race/ethnicity, SES, geographic location, histologic type, tumor size, and tumor stage. We used Joinpoint Regression software (version 5.3.0) to compute average annual percent change (AAPC) and analyze thyroid cancer trends over the pre-specified fixed intervals (2000–2009, 2010–2015, and 2016–2020), reflecting ATA revision years in 2009 and 2015. This allowed us to compare thyroid cancer trends and incidence rates before and after the implementation and revisions of the ATA guidelines. We then computed incidence rate ratios (IRRs) and used the Tiwari method to calculate 95% CIs for the IRRs [29]. Lastly, we used Joinpoint Regression software to fit, assess, and compare thyroid cancer incidence and trends over time stratified by race, SES, geographic location, histology, tumor size, and tumor stage [30]. The best-fitted line was selected using the Monte Carlo permutation method [30]. APCs were calculated to summarize the stratified trends. Furthermore, *p*-values < 0.05 were considered statistically significant for the descriptive statistics and trend analyses.

## 3. Results

An overview of the 187,964 patients diagnosed with thyroid cancer between 2000 and 2020 is presented in Table 1. Overall, NH White patients (63.5%), females (76.5%), residents of metropolitan geographic areas (90.2%), PTC (88.2%), and early-stage tumor (66.6 %) patients constituted the majority of the study cohort. NH Black patients exhibited a higher percentage of low SES (47.9%) compared with NH White (33.7%), Hispanic (36.6%), and NH other (19.5%) patients.

The AAPC from the joinpoint analysis revealed some variations in thyroid cancer incidence trends based on time, aligning with the ATA revision years (Table 2). Between 2000 and 2009, the incidence of thyroid cancer increased by 5.8% on average annually (AAPC 5.8, *p* < 0.05). Following the initial pronounced increase, thyroid cancer increased marginally (AAPC: 1.1, *p* < 0.05) between 2010 and 2015 before significantly decreasing at 4.8% annually between 2016 and 2020 (AAPC −4.8, *p* < 0.05). The IRRs of thyroid cancer between 2000 and 2020 were stratified by race/ethnicity, SES, geographic location, histology, tumor size, and tumor stage (Table 3). Between 2000 and 2020, NH Black (IRR: 0.56, 95% CI 0.55–0.57), Hispanic (IRR: 0.87, 95% CI 0.86–0.88), and NH other patients (IRR: 0.97, 95% CI 0.95–0.98), had a significantly lower age-adjusted thyroid cancer incidence rate than NH White patients. Between 2000 and 2020, the thyroid cancer incidence rate in the high-SES group was 1.13 (95% CI 1.11–1.14) times higher than that in the low-SES tertile. However, the strength of association changed when we stratified the result by the time periods reflecting ATA revision years. For instance, between 2000 and 2009, the thyroid cancer incidence rate in the high-SES group was 1.21 (95% CI 1.19–1.24) times higher than that in the low-SES tertile. The same comparison between 2010 and 2015 yielded similar results to 2000–2009. Between 2016 and 2020, however, the age-adjusted incidence rate in the high-SES group was 1.07 (95% CI 1.05–1.09) times higher than that in the low-SES tertile. Patients residing in non-metropolitan geographic areas had a lower thyroid cancer incidence rate compared with those in metropolitan areas, with an IRR of 0.90 (95% CI 0.89–0.92). The study revealed substantial variations in age-adjusted incidence rates by histologic type. The age-adjusted incidence rates for FTC (IRR: 0.085, 95% CI 0.084–0.087), MTC (IRR: 0.019, 95% CI 0.018–0.020), and ATC (IRR: 0.0099, 95% CI 0.0095–0.01) were notably lower when compared with PTC. When we stratified the histology IRRs by time periods, we observed similar results, although the magnitudes fluctuated from one time period to the next.

The results of the joinpoint trend analyses of thyroid cancer incidence stratified by race/ethnicity, SES, geographic location, histology, tumor size, and tumor stage are shown in Table 4. Overall, the thyroid cancer incidence increase was comparable between all racial groups; however, the joinpoint analysis identified segments exhibiting different trends within each racial/ethnic group. Among NH White patients, thyroid cancer incidence had a pronounced increase between 2000 and 2009 (APC: 6.8, *p* < 0.05); it plateaued between 2009 and 2015 with no significant increase, and it sharply decreased between 2015 and 2020 (APC: −4.7, *p* < 0.05). NH Black patients had an incidence increase between 2000 and 2014 (APC: 5.2, *p* < 0.05). However, between 2014 and 2020, it significantly decreased (APC: –5.0, *p* < 0.05). The trend for Hispanic patients was similar to that of NH Black patients, although the segment years and magnitudes differed. In this group, we observed an increase in incidence between 2000 and 2014 (APC: 4.5, *p* < 0.05) and a decrease (APC: −2.6, *p* < 0.05) between 2014 and 2020. The increase in thyroid cancer incidence since 2000 was more significant among PTC patients (APC: 3.3, *p* < 0.05) compared with MTC (1.0, *p* > 0.05) and ATC (1.8, *p* < 0.05) patients. FTC cases, on the other hand, showed a marginal decrease in incidence at the same time (APC: −0.8, *p* < 0.05)

**Figure 1:**
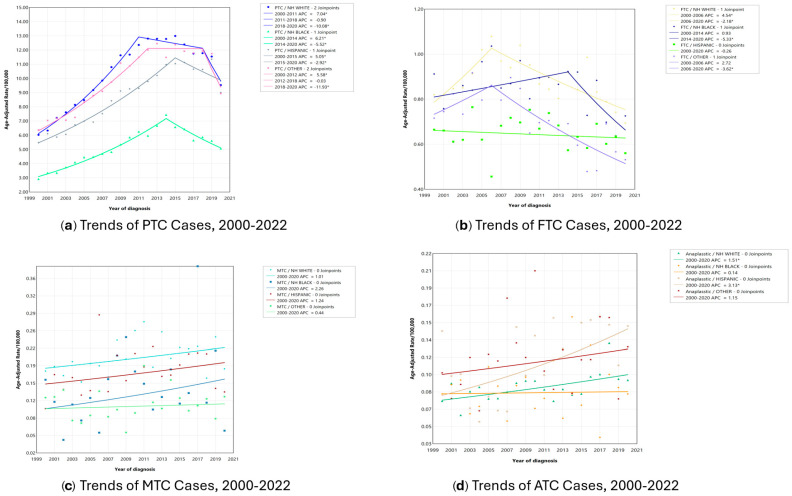
Age-adjusted incidence rate trends between 2000 and 2020 for each histologic type by race/ethnicity. * marks results with *p*-values < 0.05.

**Figure 2:**
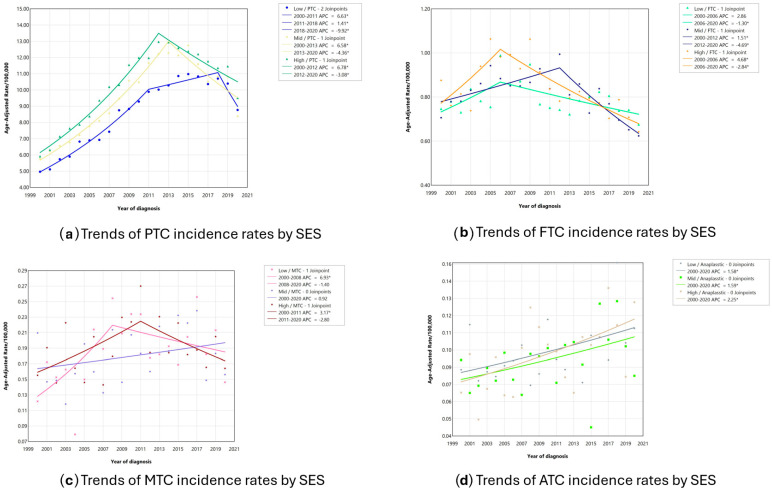
Age-adjusted incidence rate trends between 2000 and 2020 for each histologic type by SES. * marks results with *p*-values < 0.05.

Figure 1 displays the incidence rate trends between 2000 and 2020 for each histologic type stratified by racial and ethnic groups. The general trend among PTC cases is a steady increase in the first decade, followed by a sharp decrease (Figure 1a). The greatest increase in PTC incidence rate was among NH White patients between 2000 and 2011 (APC: 7.0, *p* < 0.05), followed by NH Black patients between 2000 and 2014 (APC: 6.2). The sharpest decrease in PTC incidence rate was observed between 2018 and 2020 among NH White (−10.1, *p* < 0.05) and NH Other patients (−11.9, *p* < 0.05). For NH Black (APC: −5.5, *p* < 0.05) and Hispanic patients (APC: −5.1, *p* < 0.05), the incidence of PTC decreased between 2014–2020 and 2015–2020, respectively. NH White patients were the only racial/ethnic group to experience a significant increase in FTC incidence rate (2000–2006) followed by a steady decrease over time (Figure 2b). The incidence of FTC among NH Black patients remained relatively constant between 2000 and 2014, followed by a pronounced and significant decrease between 2014 and 2020. For ATC, the incidence rate increased steadily for the entirety of the study period among NH White (APC: 1.5, *p* < 0.05) and Hispanic (APC: 3.1, *p* < 0.05) patients (Figure 1d), while the ATC incidence rate remained similar for the remaining racial/ethnic groups. The stratified MTC incidence rate by race/ethnicity showed no statistically significant changes.

The age-adjusted incidence rate trends (Figure 2) for FTC, stratified by SES, showed no statistically significant change among low-SES patients between 2000 and 2006 (Figure 2b). However, from 2006 to 2020, the FTC incidence rate decreased by 1.3% annually (APC: −1.3, *p* < 0.05). Among high-SES patients, the FTC incidence rate increased between 2000 and 2006 (APC: 4.7, *p* < 0.05) before decreasing from 2006 to 2020 (APC: −2.8, *p* < 0.05). For ATC, we observed steady increases across all SES groups, with the highest increase among high-SES patients between 2000 and 2020 (Figure 2d). Overall, the age-adjusted incidence rate trends for each histologic type, stratified by SES, showed inflection points around the years of the ATA’s guidelines implementation and/or revisions.

## 4. Discussion

This retrospective cohort study investigated the temporal, socioeconomic, histologic, geographic, and racial variations in thyroid cancer incidence from 2000 to 2020. Overall, we found that the thyroid cancer incidence rate increased steadily between 2000 and 2009, followed by a mild increase between 2010 and 2015 and a sharp decrease between 2016 and 2020. Similarly, the joinpoint analysis stratified by race/ethnicity, SES, geographic area, histology, and tumor stage consistently identified joinpoints around 2006, 2009, and 2015, coinciding with the ATA’s guidelines implementation and the two major thyroid cancer management guidelines revision years. More noticeably, NH White patients, who were more likely to be in the high-SES category, had a pronounced incidence increase until 2009, remained constant until 2015, and started decreasing in 2015. These findings suggest that thyroid cancer incidence rate was partially influenced by the thyroid cancer management recommendations amendments, especially among high-SES and NH White patients.

We also found that NH White patients had noticeably higher incidence rates than NH Black, Hispanic, and NH Other patients, aligning with the existing evidence [7]. This can be partially justified in that NH White patients often undergo more extensive testing, potentially leading to overdiagnosis compared with other racial/ethnic groups [7]. Moreover, NH White patients are generally more likely to be of a higher SES, have access to better insurance, or have better health literacy than their Black counterparts [6,10]. Our study revealed a positive dose–response association between the incidence of SES tertile and thyroid cancer. This finding is supported by Li et al. (2013), who found that, on average, patients with thyroid cancer in the US had higher SES than the average SES [5]. This is explained by the fact that people in high SES categories often have a higher level of education, health literacy, and access to cutting-edge diagnostic tools, such as high-resolution ultrasound and FNA, facilitating early detection [5,31,32,33,34]. Conversely, patients in low-SES or underserved racial/ethnic groups face barriers such as limited healthcare access, lower insurance coverage, and reduced health literacy, which may result in delayed diagnoses or underreporting of thyroid cancer cases. These disparities highlight the critical need to address inequities in healthcare access and resource distribution to improve thyroid cancer outcomes across all populations.

Consistent with the published literature [12,35,36], thyroid cancer incidence rates differed by histologic type. We found that PTC cases had a higher incidence rate, whereas ATC had the lowest incidence rate of all histologic types. Moreover, the overall trends analysis revealed that PTC, followed by ATC, had the highest annual incidence rate increases between 2000 and 2020, mainly driven by the high-SES group. PTC is the most common type of thyroid cancer globally and has a higher incidence rate among individuals from higher SES groups, who often benefit from regular check-ups and early detection [37,38,39]. In contrast, ATC, a rare and aggressive form of thyroid cancer that typically arises from more differentiated cancers like PTC that lose their differentiation, is often more prevalent in lower SES groups [33,37,38,39]. This disparity is due to limited access to healthcare in lower SES populations, leading to delayed diagnosis and treatment, often only after the cancer has progressed to an advanced stage. This trend is also observed internationally, with a higher incidence of ATC in lower-income countries such as the Democratic Republic of Congo compared with Western nations [40]. These findings underscore the critical need to address healthcare disparities by improving access and early detection efforts, especially among low-SES and minority populations, who often face worse prognoses due to late-stage diagnoses of more aggressive cancers like ATC.

The results from our study suggested that the association of race/ethnicity, SES, geographic location, and histology with thyroid cancer incidence trends may have been impacted by ATA’s thyroid cancer management revisions in 2009 and 2015 [14,15,16]. Although the overall incidence rate trend between 2000 and 2020 suggested a comparable increase within each category of the key predictors, the joinpoint analysis identified segments that displayed different rate changes. The general trend is an increase in the thyroid cancer incidence rate followed by a decrease. The most common joinpoints were in 2009 and 2015, which aligned with the ATA’s thyroid cancer management guidelines revision years. The latest iterations of the ATA’s guidelines redefined thyroid nodule risk assessment and stratifications by emphasizing the importance of biopsy based on sonographic patterns and molecular markers to reduce unnecessary biopsies and enhance the accuracy of diagnostic assessments. By reducing the number of unnecessary biopsies and enhancing the accuracy of diagnostic assessments, the revised guidelines can mitigate overdiagnosis and false positives, thereby impacting the number of thyroid cancers diagnosed and reported [14,15,16].

To the best of our knowledge, this is the first study in the US that investigated the impact of the ATA guidelines revisions in 2009 and 2015 on the thyroid cancer incidence rate over time and how thyroid cancer trends varied by histology and race. In addition, we used data from a national database (i.e., SEER), enhancing our ability to generalize our results to the US population [20]. Furthermore, we used a composite metric of SES, which bolstered our confidence in making inferences about the impact of SES on thyroid cancer trends.

The main limitation of our study is that census tracts at county-level measures were utilized to assign SES groups instead of individual SES metrics, which may have introduced some levels of ecological fallacies or biases in our obtained SES results. County-level SES data may not fully capture individual-level variations, leading to possible overgeneralizations or misinterpretations of the relationship between SES and age-adjusted incidence rates. This limitation should be considered when interpreting our findings, particularly in stratified analyses. In addition, the incidence rates were adjusted to the 2000 US standard population, which may not reflect the US population and makeup in subsequent years. Lastly, our trends analysis did not provide a contextual understanding of the underlying genetics or environmental dynamics that potentially influence the observed trends.

## 5. Conclusions

In summary, we found some intricate dynamics in thyroid cancer incidence rate trends from 2000 to 2020 that were shaped by temporal, socioeconomic, histologic, geographic, and racial/ethnic factors. The observed initial rise was followed by a decline in the thyroid cancer incidence rate, with notable joinpoints coinciding with significant revisions to the ATA’s guidelines in 2009 and 2015. The results of this study suggest that the changes in thyroid cancer incidence trends over the past two decades can be partially explained by improvements in diagnostic tools, indirectly affected through SES and thyroid cancer management recommendations.

## Figures and Tables

**Table 1:** Tumor and sociodemographic characteristics of US adults diagnosed with thyroid cancer, 2000–2020.

	Total N = 187,964 *n* (%)	NH White N = 119,296 *n* (%)	NH Black N = 11,810 *n* (%)	Hispanic N = 33,062 *n* (%)	NH Other N = 21,971 *n* (%)	Unknown N = 21,971 *n* (%)
**Sex**						
Female	143,799 (76.5)	88,422 (74.1)	9700 (82.1)	26,877 (81.3)	17,389 (79.2)	1411 (77.3)
Male	44,165 (23.5)	30,874 (25.9)	2110 (17.9)	6185 (18.7)	4582 (20.9)	414 (22.7)
**Age at diagnosis**						
<40	55,459 (29.7)	32,463 (27.3)	2983 (25.4)	12,432 (38.0)	6907 (31.6)	674 (37.1)
40–49	42,546 (22.8)	26,234 (22.1)	2799 (23.8)	7948 (24.3)	5134 (23.5)	431 (23.7)
50–59	41,804 (22.4)	27,505 (23.2)	2890 (24.6)	6405 (19.6)	4609 (21.1)	395 (21.7)
60–69	28,519 (15.3)	19,487 (16.4)	1998 (17.0)	3668 (11.2)	3153 (14.4)	213 (11.7)
70–79	14,030 (7.5)	9834 (8.3)	836 (7.1)	1747 (5.3)	1528 (7.0)	85 (4.7)
80+	4583 (2.5)	3242 (2.7)	251 (2.1)	539 (1.7)	532 (2.4)	19 (1.1)
**SES**						
Low	62,794 (33.4)	40,260 (33.7)	5652 (47.9)	12,086 (36.6)	4293 (19.5)	503 (27.6)
Middle	62,963 (33.5)	39,308 (32.9)	3930 (33.3)	12,715 (38.5)	6387 (29.1)	623 (34.1)
High	62,175 (33.1)	39,713 (33.3)	2227 (18.9)	8250 (25.0)	11,290 (51.4)	695 (38.1)
Unknown	28 (0.0)	15 (0.0)	1 (0.0)	7 (0.0)	1 (0.0)	4 (0.2)
**Geographic Area**						
Metropolitan	169,571 (90.2)	104,039 (87.2)	10,838 (91.8)	31,913 (96.5)	21,047 (95.8%)	1734 (95.0%)
Non-Metropolitan	18,157 (9.7)	15,242 (12.8)	971 (8.2)	1138 (3.4)	719 (3.3)	87 (4.8)
Unknown	236 (0.1)	15 (0.0)	1 (0.0)	11 (0.0)	205 (0.9)	4 (0.2)
**Marital status**						
Married/Domestic Partner	113,869 (60.6)	75,479 (63.3)	4774 (40.4)	18,262 (55.2)	14,535 (66.2)	819 (44.9)
Divorced/Separated	14,380 (7.7)	9341 (7.8)	1354 (11.5)	2553 (7.7)	1047 (4.8)	85 (4.7)
Widowed	8912 (4.7)	5749 (4.8)	738 (6.3)	1273 (3.9)	1114 (5.1)	38 (2.1)
Never Married	40,507 (21.6)	22,554 (18.9)	4191 (35.5)	9221 (27.9)	4193 (19.1)	348 (19.1)
Unknown	10,296 (5.5)	6173 (5.2)	753 (6.4)	1753 (5.3)	1082 (4.9)	535 (29.3)
**Tumor stage**						
Early	125,214 (66.6)	82,804 (69.4)	9256 (78.4)	19,029 (57.6)	12,955 (59.0)	1170 (64.1)
Advanced	58,983 (31.4)	34,420 (28.9)	2304 (19.5)	13,328 (40.3)	8528 (38.8)	403 (22.1)
Unknown	3767 (2.0)	2072 (1.7)	250 (2.1)	705 (2.1)	488 (2.2)	252 (13.8)
**Histology**						
PTC ^a^	165,750 (88.2)	104,797 (87.9)	9606 (81.3)	29,823 (90.2)	19,909 (90.6)	1615 (88.5)
FTC ^b^	14,223 (7.6)	9325 (7.8)	1488 (12.6)	1973 (6.0)	1312 (6.0)	125 (6.9)
MTC ^c^	3252 (1.7)	2206 (1.9)	269 (2.3)	534 (1.6)	215 (1.0)	28 (1.5)
ATC ^d^	1685 (0.9)	1092 (0.9)	131 (1.1)	250 (0.8)	208 (1.0)	4 (0.2)
Other	3054 (1.6)	1876 (1.6)	316 (2.7)	482 (1.5)	327 (1.5)	53 (2.9)
**Tumor size**						
0–1	63,338 (33.7)	43,325 (36.3)	4024 (34.1)	8909 (27.0)	6491 (29.5)	589 (32.3)
1.1–2	50,373 (26.8)	31,937 (26.8)	2369 (20.1)	9251 (28.0)	6390 (29.1)	426 (23.3)
2.1–4	43,306 (23.0)	26,050 (21.8)	2635 (22.3)	8837 (26.7)	5447 (24.8)	337 (18.5)
4+	19,937 (10.6)	11,332 (9.5)	1955 (16.6)	4078 (12.3)	2434 (11.1)	138 (7.6)
Unknown	11,010 (5.9)	6652 (5.6)	827 (7.0)	1987 (6.0)	1209 (5.5)	335 (18.4)
**Year of diagnosis**						
2000–2009	70,287(37.4)	47,768 (40.0)	4200 (35.6)	10,433 (31.6)	7449 (33.9)	437 (23.9)
2010–2015	64,622 (34.4)	40,790 (34.2)	4185 (35.4)	11,372 (34.4)	7648 (34.8)	627 (34.4)
2016–2020	53,055 (28.2)	30,738 (25.8)	3425 (29.0)	11,257 (34.4)	6874 (31.3)	761 (41.7)

Pearson’s chi-square identified all the associations above as significant. a: Papillary thyroid cancer. b: Follicular thyroid cancer. c: Medullary thyroid cancer. d: Anaplastic thyroid cancer.

**Table 2:** Thyroid cancer average annual percent change by time period.

	Thyroid Cases Diagnosed N	Total Population at Risk N	Age-Adjusted Incidence Rate per 100,000 (95% CI)	AAPC (95% CI)
**Time Period**				
2000–2009	70,287	782,534,156	9.0 (8.9,9.1)	5.8 (5.3,6.5) *
2010–2015	64,622	502,910,809	12.5 (12.4,12.6)	1.1 (0.2,1.9) *
2016–2020	53,055	432,870,330	11.8 (11.7,11.9)	−4.8 (−6.1,−3.4) *
2000–2020	187,964	1718,315,295	10.7 (10.6,10.8)	2.1 (1.8,2.5) *

*: *p* < 0.05.

**Table 3:** Thyroid cancer incidence rate ratio stratified by race/ethnicity, SES, geographic area, histology, and tumor size, 2000–2020.

	2000–2020		2000–2009		2010–2015		2016–2020	
	Age-Adjusted Incidence Rate per 100,000 95% CI	Incidence Rate Ratio 95% CI	Age-Adjusted Incidence Rate per 100,000 95% CI	Incidence Rate Ratio 95% CI	Age-Adjusted Incidence Rate per 100,000 95% CI	Incidence Rate Ratio 95% CI	Age-Adjusted Incidence Rate per 100,000 95% CI	Incidence Rate Ratio 95% CI
**Race/Ethnicity**								
NH White	11.7 (11.6,11.7)	1.00 (Ref)	9.9 (9.8,10.0)	1.00 (Ref)	13.9 (13.8,14.1)	1.00 (Ref)	12.7 (12.6,12.9)	1.00 (Ref)
NH Black	6.5 (6.4,6.6)	0.56 (0.55–0.57) *	5.4 (4.2,5.6)	0.55 (0.53–0.56) *	7.8 (7.5,8.0)	0.56 (0.54–0.58) *	6.9 (6.7,7.1)	0.55 (0.53–0.57) *
Hispanic	10.1 (10.0,10.3)	0.87 (0.86–0.88) *	8.1 (7.9,8.3)	0.82 (0.80–0.84) *	11.3 (11.1,11.5)	0.81 (0.79–0.83) *	11.7 (11.5,11.9)	0.91 (0.90–0.94) *
Other	11.3 (11.1,11.4)	0.97 (0.95–0.98) *	9.5 (9.3,9.8)	0.96 (0.94–0.99) *	12.9 (12.6–13.2)	0.93 (0.90–0.95) *	12.2 (11.5,11.9)	0.96 (0.93–0.99)
**SES**								
Low	10.2 (10.2,10.3)	1.00 (Ref)	8.0 (7.9,8.2)	1.00 (Ref)	11.5 (11.3,11.6)	1.00 (Ref)	11.6 (11.4,11.7)	1.00 (Ref)
Middle	10.5 (10.5,10.6)	1.03 (1.02–1.04) *	9.0 (8.9,9.1)	1.12 (1.10–1.14) *	13.2 (12.9,13.4)	1.15 (1.13–1.17) *	11.5 (11.3,11.7)	0.99 (0.97–1.017)
High	11.5 (11.4,11.6)	1.13 (1.11–1.14) *	9.8 (9.7,9.9)	1.21 (1.19–1.24) *	13.8 (13.6,13.9)	1.20 (1.18–1.23) *	12.4 (12.2,12.6)	1.07 (1.05–1.09) *
**Geographic area**								
Metropolitan	10.9 (10.8,10.9)	1.00 (Ref)	9.1 (9.1,9.2)	1.00 (Ref)	12.7 (12.6,12.8)	1.00 (Ref)	11.8 (11.7,11.9)	1.00 (Ref)
Non-Metropolitan	9.8 (9.7,10.0)	0.90 (0.89–0.92) *	8.1 (7.9,8.3)	0.89 (0.87–0.91) *	11.5 (11.2,11.8)	0.91 (0.88,0.93) *	11.5 (11.1,11.9)	0.97 (0.94–1.01)
**Histology**								
PTC ^a^	9.5 (9.4,9.5)	1.00 (Ref)	7.7 (7.6,7.8)	1.00 (Ref)	11.3 (11.2,11.4)	1.00 (Ref)	10.6 (10.5,10.7)	1.00 (Ref)
FTC ^b^	0.8 (0.7,0.8)	0.085 (0.084–0.087) *	0.9 (0.8,0.9)	0.112 (0.109,0.115) *	0.8 (0.7,0.8)	0.071 (0.069–0.074) *	0.7 (0.7,0.8)	0.069 (0.067–0.072) *
MTC ^c^	0.2 (0.2,0.2)	0.019 (0.018–0.020) *	0.2 (0.2,0.2)	0.022 (0.021–0.023) *	0.2 (0.2,0.2)	0.019 (0.017,0.019) *	0.2 (0.2,0.2)	0.018 (0.017–0.019) *
ATC ^d^	0.1 (0.1,0.1)	0.010 (0.009–0.010) *	0.1 (0.1,0.1)	0.010 (0.010–0.012) *	0.1 (0.1,0.1)	0.008 (0.007–0.009) *	0.1 (0.1,0.1)	0.010 (0.009–0.11) *
Other	0.2 (0.2,0.2)	0.021 (0.021–0.022) *	0.2 (0.2,0.2)	0.025 (0.024–0.026) *	0.2 (0.2,0.2)	0.018 (0.017–0.019) *	0.2 (0.2,0.2)	0.021 (0.019–0.022) *
**Tumor Size**								
0–1	3.6 (3.5,3.6)	1.00 (Ref)	2.9 (2.8,2.9)	1.00 (Ref)	4.6 (4.5,4.6)	1.00 (Ref)	3.8 (3.7,3.8)	1.00 (Ref)
1.1–2	2.9 (2.8,2.9)	0.80 (0.79–0.81) *	2.3 (2.2,2.3)	0.79 (0.78–0.81) *	3.4 (3.3,3.4)	0.74 (0.72–0.76) *	3.4 (3.3,3.4)	0.90 (0.88–0.92) *
2.1–4	2.5 (2.5,2.5)	0.69 (0.67–0.70) *	2.1 (2.1,2.2)	0.73 (0.72–0.75) *	2.8 (2.7,2.8)	0.61 (0.59–0.61) *	2.8 (2.8,2.9)	0.76 (0.74–0.77) *
4+	1.1 (1.1,1.2)	0.32 (0.31–0.32 *	0.9 (0.9,1.0)	0.32 (0.21–0.33) *	1.3 (1.2,1.3)	0.28 (0.27–0.28)	1.4 (1.3,1.4)	0.36 (0.35–0.38) *
**Tumor stage**								
Early	7.1 (7.1,7.2)	1.00 (Ref)	6.1 (6.0,6.2) *	1.00 (Ref)	8.4 (8.3,8.4)	1.00 (Ref)	7.6 (7.5,7.7)	1.00 (Ref)
Advanced	3.4 (3.4,3.5)	0.47 (0.46–0.48) *	2.7 (2.7,2.7) *	0.44 (0.43,0.45)	3.9 (3.9,4.0)	0.47 (0.46–0.48) *	4.0 (3.9,4.1)	0.52 (0.51–0.53) *

Ref: Reference group. * indicates that the annual percent change (APC) is significantly different from 0 at alpha = 0.05. a: Papillary thyroid cancer. b: Follicular thyroid cancer. c: Medullary thyroid cancer. d: Anaplastic thyroid cancer.

**Table 4:** Thyroid cancer incidence trends by race, SES, histology, and tumor size, 2000–2020.

	Joinpoint Analysis: 2000–2020									
	Overall Trend		Segment 1		Segment 2		Segment 3		Segment 4	
	Years	APC (CI)	Years	APC1 (CI)	Years	APC2 (CI)	Years	APC3 (CI)	Years	APC4 (CI)
**Race/Ethnicity**										
NH White	2000–2020	2.8 (1.5,4.1) *	2000–2009	6.8 (6.0,7.9) *	2009–2015	1.8 (−0.1,3.7)	2015–2020	−4.7 (−7.1,−3.2) *		
NH Black	2000–2020	2.8 (1.6,3.9) *	2000–2014	5.2 (4.6,5.8) *	2014–2020	−5.0 (−7.3,−3.1) *				
Hispanic	2000–2020	3.3 (2.4,4.1) *	2000–2015	4.5 (3.9,5.2) *	2015–2020	−2.6 (−6.6,−0.1)				
Other	2000–2020	2.6 (1.5,3.6) *	2000–2012	4.9 (4.3–6.0) *	2012–2018	−0.3 (−2.0,3.0)	2018–2020	−11.0 (−15.4,−4.7)		
**SES**										
Low	2000–2020	3.4 (2.4,4.4) *	2000–2011	5.8 (5.2,6.7) *	2011–2018	1.2 (−0.1,2.8)	2018–2020	−8.9 (−13.2,−3.8) *		
Middle	2000–2020	2.8 (1.3,4.2) *	2000–2013	5.8 (5.1,6.6) *	2013–2020	−4.2 (−6.2,−2.6) *				
High	2000–2020	2.7 (1.3,4.1) *	2000–2009	6.8 (4.0,10.2) *	2009–2013	2.3 (0.2,9.2) *	2013–2018	−2.0 (−3.3,2.8)	2018–2020	−7.7 (−11.3,−3.3) *
**Area of residence**										
Metropolitan	2000–2020	2.8 (1.6,4.0) *	2000–2011	5.8 (5.3,6.5) *	2011–2018	−0.3 (−1.4,−1.3) *	2018–2020	−9.6 (−13.3,−4.7) *		
Non-Metropolitan	2000–2020	3.3 (2.2,4.5) *	2000–2009	6.1 (5.3,8.7) *	2009–2016	2.7 (0.7,4.4)	2016–2020	4.0 (−8.5,1.5) *		
**Histology**										
PTC ^a^	2000–2020	3.3 (2.0–4.5) *	2000–2011	6.6 (6.0,7.3) *	2011–2018	−0.1 (−1.2,1.5)	2018–2020	−10.1 (−13.8,−4.9) *		
FTC ^b^	2000–2020	−0.8 (−1.6,−0.1) *	2000–2006	3.9 (1.7,7.8) *	2006–2020	−2.1 (−2.9,−1.5) *				
MTC ^c^	2000–2020	1.0 (−0.1,2.1)	2000–2017	1.9 (0.3,13.3) *	2017–2020	−9.4 (−26.3,1.1)				
ATC ^d^	2000–2020	1.8 (0.7–2.8) *								
Other	2000–2020	1.0 (−0.2,2.2)								
**Tumor Size**										
0–1	2000–2020	2.9 (0.7,5.6) *	2000–2010	9.3 (8.4,10.5) *	2010–2015	1.1 (−1.2,3.6)	2015–2020	−7.7 (−10.1,−6.0)		
1.1–2	2000–2020	3.5 (2.3,4.9) *	2000–2011	7.4 (6.2,9.2) *	2011–2020	−0.5 (−2.1,0.8)				
2.1–4	2000–2020	2.6 (1.9,3.3) *	2000–2015	3.7 (3.2,4.6) *	2015–2020	−2.3 (−6.6,0.1)				
4+	2000–2020	3.2 (2.6,3.9) *	2000–2009	5.8 (4.4,9.1) *	2009–2020	1.8 (0.3,2.6) *				
**Tumor stag**										
Early	2000–2020	2.3 (0.9,3.8) *	2000–2011	5.8 (5.1,6.8) *	2011–2018	−1.9 (−1.9,4.0)	2018–2020	−8.4 (−12.2,−2.7) *		
Advanced	2000–2020	3.4 (2.2,4.8) *	2000–2009	6.6 * (5.6,10.0) *	2009–2016	3.3 (0.7,4.8) *	2016–2020	−5.0 (−9.1,−2.7) *		

* indicates that the annual percent change (APC) is significantly different from 0 at alpha = 0.05. a: Papillary thyroid cancer. b: Follicular thyroid cancer. c: Medullary thyroid cancer. d: Anaplastic thyroid cancer.

## Data Availability

The data generated and/or analyzed during the current study are not publicly available due to SEER’s data use agreement and terms. The data access requests should be made directly to SEER. https://seerdataaccess.cancer.gov/seer-data-access. (accessed on 16 September 2023). The code generated and used during this study is available upon request.

## Abbreviations

AAPC	Average annual percent change
APC	Annual percent change
ATA	American Thyroid Association
ATC	Anaplastic thyroid cancer
CI	Confidence interval
FNA	Fine-needle aspiration
FTC	Follicular thyroid cancer
IRR	Incidence rate ratio
MTC	Medullary thyroid cancer
NH	Non-Hispanic
PTC	Papillary thyroid cancer
SEER	Surveillance, Epidemiology, and End Results
SES	Socioeconomic status
US	United States
